# Novel tree-based method to generate markers from rare variant data

**DOI:** 10.1186/1753-6561-5-S9-S102

**Published:** 2011-11-29

**Authors:** Yuan Jiang, Jennifer S Brennan, Rose Calixte, Yunxiao He, Epiphanie Nyirabahizi, Heping Zhang

**Affiliations:** 1 Department of Epidemiology and Public Health, Yale School of Public Health, School of Medicine, Yale University, 60 College Street, PO Box 208034, New Haven, CT 06520-8034, USA

## Abstract

Existing methods for analyzing rare variant data focus on collapsing a group of rare variants into a single common variant; collapsing is based on an intuitive function of the rare variant genotype information, such as an indicator function or a weighted sum. It is more natural, however, to take into account the single-nucleotide polymorphism (SNP) interactions informed directly by the data. We propose a novel tree-based method that automatically detects SNP interactions and generates candidate markers from the original pool of rare variants. In addition, we utilize the advantage of having 200 phenotype replications in the Genetic Analysis Workshop 17 data to assess the candidate markers by means of repeated logistic regressions. This new approach shows potential in the rare variant analysis. We correctly identify the association between gene *FLT1* and phenotype Affect, although there exist other false positives in our results. Our analyses are performed without knowledge of the underlying simulating model.

## Background

Recent work supports the involvement of rare variants in complex disease etiology [1-3]; despite a low frequency of occurrence, rare variants may be functionally important and may account for a detectable increase in the relative risk of developing the outcome. Next-generation sequencing has great potential for important applications in human genetics, including the detection of rare variants. Several methods have been proposed to handle rare variants in association analyses [4-7]. The aim of these methods is to construct a set of markers from the original single-nucleotide polymorphisms (SNPs), using predefined groups, such as genes or nearby genomic regions. These candidate markers are then considered in an association analysis.

For example, a key analytical tool is the collapsing method. Named for the collapsing of genotypes across variants, the collapsing method uses a rare variant indicator for each subject to describe the presence of rare variants in prespecified subsets of SNPs. Li and Leal [5] extended the collapsing method to the framework of multiple-marker tests, and called their method the combined multivariate and collapsing (CMC) method. They proposed dividing the markers into subgroups according to specific criteria and then collapsing the genotypes within each group. Morris and Zeggini’s [7] collapsing method uses an indicator variable for the presence of the rare variants and a quantitative variable for the proportion of the variants that carry at least one copy of the minor allele. Madsen and Browning [6] developed a weighted-sum statistic as a groupwise association test for rare variants. Their method constructs markers by taking a weighted sum of the number of rare variants from a subset of SNPs. The weights depend on the mutation frequency in the unaffected individuals. Although these methods are intuitive and easy to implement, they construct the markers without considering SNP interactions.

The goal of our paper is to develop an approach that constructs meaningful markers generated from the data. As in previous methods, we aim to define a single marker for a set of SNPs. However, our framework focuses on detecting the interaction among these SNPs by fitting a phenotype association model. Interaction among a group of SNPs describes the situation in which the joint influence of this group on the response variable is not additive. Because most of the SNPs are rare, a regression model including explicit interactions is unaffordable and lacks power. Thus we use the recursive partitioning method to automatically search the interactions between the SNPs in an explicit way. Once interactions are detected among a group of SNPs, we can define a marker specifically by these interactions.

## Methods

### Preprocessing

We assessed deviations from Hardy-Weinberg equilibrium (HWE) for common variants [8]. All SNPs with a minor allele frequency (MAF) greater than 0.05 that failed the HWE test (*p* < 0.0001) were excluded from further analysis.

We conducted an analysis to identify population stratification using PLINK [9]. We then computed a similarity matrix based on the pairwise identity-by-state (IBS) distances. Next we performed a multidimensional scaling (MDS) analysis on the similarity matrix. Finally, we used the pam function (a modified, more robust construction of *K* means) in the R package to perform a cluster analysis with the terminal cluster number set to 3.

### Combining SNPs into markers using recursive partitioning

Statistical interaction describes the situation in which the simultaneous influence of a group of explanatory variables on the response variable is not additive. Recursive partitioning is a natural nonparametric approach to modeling interactions among a group of variables [10,11]. Ultimately, the recursive partitioning framework meaningfully groups the rare variants into what are essentially common variants. Depending on the format of a phenotype, a classification or regression tree can be applied. For the sake of clarity, we consider only the simplest case: a binary classification tree for a binary response.

**Figure 1:**
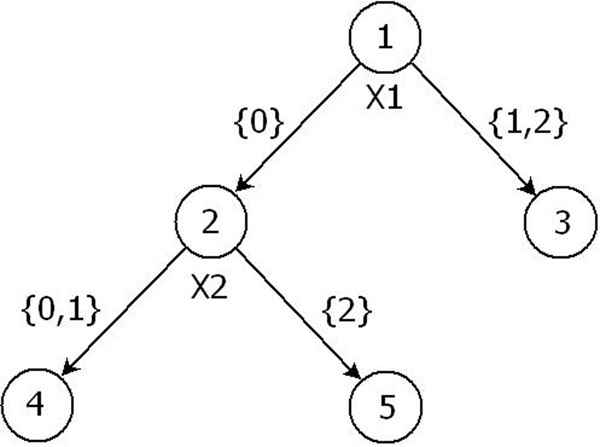
**Example tree.** An example tree fitted using phenotype *Y* and a group of SNP variables *X*_1_, *X*_2_, *X*_3_, and *X*_4_. *X*_1_ and *X*_2_ are used as the partitioning variables, yielding three leaf nodes. *X*_1_, *X*_2_, *X*_3_, and *X*_4_ are the searched SNPs, and *X*_1_ and *X*_2_ are the final SNPs used in the tree. The branches of this tree are grouped into two categories depending on the prediction value (case or control) of each leaf node. A bilevel marker is then constructed according to this categorization of branches.

We used the first replication of the phenotype in the Genetic Analysis Workshop 17 (GAW17) data to construct the classification tree. Here, Affected, denoted by *Y*, is the binary outcome (case or control, or 1 or 0) used as the response to construct the classification tree. A single binary classification tree can be grown for all SNPs within a shared gene. The tree grows as a result of recursively partitioning a node by a single SNP into two daughter nodes. Because we consider a SNP to be a three-level factor variable *X*, with levels 0, 1, and 2, the partitioning occurs when *X* is partitioned as {0} and {1, 2} or as {0, 1} and {2}. Once a tree model is built, each terminal leaf node contains a set of observations, which are predicted as case subjects or control subjects (1 or 0). This terminal node takes on the value of the outcome majority of observations in that node. We illustrate the construction of a marker using the example tree model in Figure 1.

Suppose that the example tree shown in Figure 1 is fitted using phenotype *Y* and a group of SNP variables *X*_1_, *X*_2_, *X*_3_, and *X*_4_. Let’s consider the case that *X*_1_ and *X*_2_ are used as the partitioning variables (thus *X*_1_, *X*_2_, *X*_3_, and *X*_4_ are the searched SNPs and *X*_1_ and *X*_2_ are the final SNPs used in the tree). Accordingly, the tree indicates an interaction between *X*_1_ and *X*_2_. Suppose that the leaf nodes 3 and 4 contain a majority of “control” subjects; these two leaf nodes are then also considered “control” leaves. Similarly, if leaf node 5 contains a majority of “case” subjects, then node 5 is considered a “case” leaf. The path to reach each of these three leaf nodes from the initial root is described by the tree’s three branches: {*B*_1_: *X*_1_ > 0}, {*B*_2_: *X*_1_ = 0 and *X*_2_ < 2}, {*B*_3_: *X*_1_ = 0 and *X*_2_ = 2}. Thus the branches *B*_1_ and *B*_2_ have the same effect direction because they both result in a “control” prediction. *B*_3_, however, has the reverse effect direction because it results in a “case” prediction. Thus a bilevel marker can be constructed: one level of the marker represents *B*_1_ and *B*_2_, and the other level of the marker represents *B*_3_. It is noteworthy that this bilevel marker is exactly the same as the fitted value of *Y* for all of the subjects.

For the GAW17 data set, each SNP belongs to one and only one gene, thus providing a natural set of groups, each of which can generate a tree. Therefore each marker is defined using the fitted value of *Y* for each gene, so long as the marker is not rare (our threshold was 1 − 0.99^2^ = 0.0199). Many genes, however, had a very small number of SNPs (e.g., 1,226 genes had only 1 SNP and 440 genes had 2 SNPs). For these genes with few typed SNPs, a tree can still be built using the sparse SNPs. However, use of such SNPs in trees still generates markers that have rare minor alleles. This motivates the following two-step procedure to construct the markers on a chromosome:

**Step 1.** For each gene on the chromosome, build a tree model for the phenotype Affected using the SNPs contained in that gene. Record a single marker for each gene if the minor allele of the marker is not rare.

**Step 2.** For all the remaining SNPs not considered in step 1, perform a clustering analysis to cluster nearby SNPs on the chromosome. If the minor allele of the marker is not rare, consider this marker in tree construction from each cluster of SNPs.

### Evaluating the markers by repeated independent logistic regressions

We used only the first replication of the phenotype Affected to construct the markers; the remaining 199 phenotype replications were used to evaluate these markers. To evaluate each marker, we fitted a logistic regression using Affected as the response variable against each marker, adjusted for sex, age, smoking status, and race (ethnicity) cluster covariates.

For each marker, there are 199 *p*-values resulting from the 199 independent tests. To assess the relative importance of each marker, we report the number of replications in which the marker achieves significance using a Bonferroni correction (*p* < 0.05/570). As a comparison, we make use of a false discovery rate (FDR) control [12] and report again the number of significant replications.

## Results and discussion

Of the 24,487 SNPs, 87.2% (*n* = 21,355) have MAF < 0.05, 74.0% (*n* = 18,131) have MAF < 0.01, and 38.5% (*n* = 9,433) have MAF < 0.001. Those MAFs that are less than 0.001 are all equal to 0.000717. Because we have 697 unrelated individuals, 9,433 SNPs have a single minor allele across the whole sample, indicating that the coded variables for these 9,433 SNPs are a vector with 696 0’s and a single 1. This is quite problematic from a statistical point of view. Both univariate and multivariate association analyses are drastically underpowered and will not directly work for these particularly sparse variables.

We conducted an analysis to identify population stratification and to produce a similarity matrix based on the pairwise IBS distances. We then used the similarity matrix to perform an MDS analysis. The plot of the first two MDS dimensions reveals a clear clustering structure of three distinct groups. The three resulting clusters from the pam function in R almost identically match the population structure, and three homogeneous ethnic groups can be identified (Table 1).

**Table 1:** Populations and corresponding clusters

Population	Cluster		
	
	1	2	3
CEPH-1	45	0	0
CEPH-2	44	1	0
Tuscan	62	0	0
Tuscan, additional	4	0	0
Denver Chinese	0	87	0
Denver Chinese, additional	0	20	0
Han Chinese 1	0	25	0
Han Chinese 2	0	36	0
Han Chinese, additional	0	48	0
Japanese 1	0	31	0
Japanese 2	0	41	0
Japanese, additional	0	33	0
Luhya	0	0	90
Luhya, additional	0	0	18
Yoruba 1	0	0	40
Yoruba 2	0	0	47
Yoruba, additional	0	0	25

**Figure 2:**
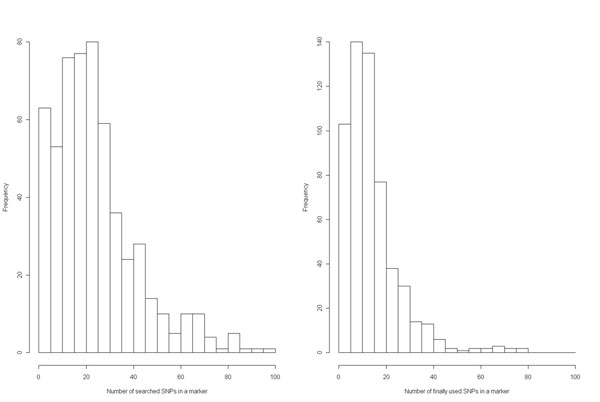
**SNP numbers that were searched (left panel) and finally used (right panel) in the 570 markers.** The left-hand panel indicates that there were relatively few cases with more than 100 searched SNPs; thus we omit these in the plot for a more clear comparison. The right-hand panel indicates the SNPs that were used to split the tree. Note that most of the 570 markers consist of fewer than 30 SNPs, which simplifies the interpretation of the generated markers.

We identified 570 genome-wide markers after our two-step recursive partitioning method was carried out. Thus the final marker set is composed of markers from both the analysis considering SNPs in each gene separately and the analysis considering the combination of nearby SNPs in each cluster. Figure 2 provides two histograms of SNPs in the tree construction. The left-hand panel presents the number of SNPs that were searched by recursive partitioning. The right-hand panel presents the number of SNPs that were ultimately used in the tree when constructing each marker from a pool of SNPs (see the example tree in Figure 1). It is obvious that the number of searched SNPs must be greater than the number of finally used SNPs for each marker. It is noteworthy that most of the 570 generated markers consist of fewer than 30 SNPs, and the modest number of involved SNPs in each constructed marker simplifies the interpretation of the marker.

For each of the 570 constructed markers, we have 199 *p*-values from 199 replications. We report the number of replications in which the marker achieves the Bonferroni-corrected significance level (*p* < 0.05/570). Table 2 provides the 10 most frequently significant markers. The top marker, significant in 10 (out of 199) replicates, is derived from a tree that includes only the SNPs in the gene *FLT1*, which is a true signal [13]. This positive result demonstrates the potential of combining SNPs according to their interactions. The remaining nine most frequently significant markers, however, are all false positives.

**Table 2:** Ten most frequently significant markers (Bonferroni correction)

Marker	Chromosome	Number of SNPs	Frequency (%)	Included genes
405	13	18	10	*FLT1*
529	19	39	8	*CYP4F22*, *CYP4F8*, *CYP4F3*, *CYP4F12*, *OR10H3*, *OR10H5*, *OR10H1*, *LOC646610*, *TPM4*
528	19	30	5	*ZNF627*, *HSZFP36*, *ZNF440*, *ZNF700*, *ZNF763*, *LOC100129837*, *LOC729747*, *ZNF20*, *ZNF625*, *LOC730651*, *LOC100129686*, *ZNF563*, *ZNF564*, *ZNF490*, *ZNF791*
207	7	10	5	*LOC100129126*, *LOC728927*, *ZNF680*, *ZNF107*, *ZNF138*, *ZNF138*, *ZNF273*, *ZNF273*, *ZNF92*, *GUSB*
439	16	21	4	*TPSD1*
296	10	53	4	*TACC2*
533	19	13	3	*LOC100130108*, *ZNF99*, *LOC646864*, *LOC645118*, *LOC100129543*
363	11	65	3	*TYR*, *C11ORF75*, *MTMR2*, *TMEM133*, *MMP20*, *MMP27*, *CASP4*, *AASDHPPT*, *CUL5*, *ATM*, *LOC100128794*, *C11ORF53*, *BTG4*, *LAYN*, *PPP2R1B*, *TTC12*, *REXO2*, *FAM55B*, *BUD13*, *LOC283152*, *CBL*, *MFRP*, *USP2*, *TECTA*, *SORL1*, *PMP22CD*, *OR10S1*, *OR8G2*, *OR8D1*, *OR8D2*, *OR8B4*, *OR8B8*, *OR8A1*, *VSIG2*, *ESAM*, *CHEK1*, *PUS3*, *CDON*, *SRPR*, *DCPS*, *SNX19*, *HNT*
361	11	79	3	*RIC8A*, *OR10A2*, *ZNF214*, *NLRP14*, *PPFIBP2*, *CYB5R2*, *OR10A6*, *RPL27A*, *ASCL3*, *RNF141*, *TEAD1*, *PIK3C2A*, *KCNJ11*, *USH1C*, *SERGEF*, *MRGPRX4*, *SAA4*, *SAA2*, *BBOX1*, *FSHB*, *KIAA1542*, *POLR2L*, *TSPAN4*, *MUC2*, *MUC5AC*, *SLC22A18*, *OR52K1*, *OR52M1*, *OR51E1*, *OR51E2*, *OR51G1*, *OR51L1*, *OR52E2*, *HBB*, *SIRT3*, *OR51B2*, *OR51B5*, *OR51B6*, *OR51Q1*, *OR51I1*, *OR51I2*, *UBQLN3*, *UBQLNL*, *OR52B6*, *TRIM6*, *TRIM5*, *OR52N1*, *OR52N2*, *OR52E6*, *OR52E8*, *OR52E4*, *LOC390084*, *OR56A4*, *OR56A1*, *FXC1*, *C11ORF47*, *OR2AG2*, *OR2AG1*, *OR6A2*
83	2	76	3	*COL6A3*

In addition to the results in Table 2, Table 3 reports information on the 10 most frequently significant markers, as adjusted by the FDR control. Compared with Table 2, we find that the frequency (17 out of 199) of significant markers appearing in the 199 replicates is higher for the marker derived from the gene *FLT1*. The FDR control method increases the chance of detecting *FLT1* but also increases type I errors for other false-positive markers. Six markers (405, 529, 528, 207, 439, and 361) overlap in both tables. There are four disagreements in each table, indicating the differences caused by different multiple testing adjustments.

**Table 3:** Ten most frequently significant markers (FDR control)

Marker	Chromosome	Number of SNPs	Frequency (%)	Included Genes
529	19	39	18	*CYP4F22*, *CYP4F8*, *CYP4F3*, *CYP4F12*, *OR10H3*, *OR10H5*, *OR10H1*, *LOC646610*, *TPM4*
405	13	18	17	*FLT1*
528	19	30	11	*ZNF627*, *HSZFP36*, *ZNF440*, *ZNF700*, *ZNF763*, *LOC100129837*, *LOC729747*, *ZNF20*, *ZNF625*, *LOC730651*, *LOC100129686*, *ZNF563*, *ZNF564*, *ZNF490*, *ZNF791*
439	16	21	9	*TPSD1*
471	17	13	8	*CDC27*
361	11	79	8	*RIC8A*, *OR10A2*, *ZNF214*, *NLRP14*, *PPFIBP2*, *CYB5R2*, *OR10A6*, *RPL27A*, *ASCL3*, *RNF141*, *TEAD1*, *PIK3C2A*, *KCNJ11*, *USH1C*, *SERGEF*, *MRGPRX4*, *SAA4*, *SAA2*, *BBOX1*, *FSHB*, *KIAA1542*, *POLR2L*, *TSPAN4*, *MUC2*, *MUC5AC*, *SLC22A18*, *OR52K1*, *OR52M1*, *OR51E1*, *OR51E2*, *OR51G1*, *OR51L1*, *OR52E2*, *HBB*, *SIRT3*, *OR51B2*, *OR51B5*, *OR51B6*, *OR51Q1*, *OR51I1*, *OR51I2*, *UBQLN3*, *UBQLNL*, *OR52B6*, *TRIM6*, *TRIM5*, *OR52N1*, *OR52N2*, *OR52E6*, *OR52E8*, *OR52E4*, *LOC390084*, *OR56A4*, *OR56A1*, *FXC1*, *C11ORF47*, *OR2AG2*, *OR2AG1*, *OR6A2*
207	7	10	8	*LOC100129126*, *LOC728927*, *ZNF680*, *ZNF107*, *ZNF138*, *ZNF138*, *ZNF273*, *ZNF273*, *ZNF92*, *GUSB*
44	1	38	8	*FNDC7*, *STXBP3*, *CELSR2*, *MYBPHL*, *SORT1*, *CYB561D1*, *AMPD2*, *CSF1*, *KCNC4*
525	19	20	7	*ZNF554*, *ZNF555*, *ZNF556*, *ZNF57*, *ZNF77*
461	17	17	7	*KCNJ12*

## Conclusions

The primary goal in handling rare variants in association studies is to transition the data from rare to common variants. We used the first phenotype replication to identify interactions among SNPs on the same chromosome, using tree-based methods. Markers were then naturally defined from the detected interactions. Compared with previous work, this novel approach produces meaningful markers that are informed directly by the data, instead of combining groups of SNPs without considering interactions. The other 199 replications of phenotypes were then used to evaluate the set of constructed markers. We report the 10 most frequently significant markers in the 199 replications. The significance threshold was adjusted by either Bonferroni correction or FDR control.

In addition to considering the binary phenotype Affected used in our work, we can also consider the continuous phenotypes (Q1, Q2, or Q4) in the regression tree framework. The constructed markers would not necessarily be bilevel. Instead, the constructed markers could be multilevel, according to the classification of terminal nodes. We make use of the replicates to evaluate the performance of the proposed method. In real data analysis, the goal is to apply, not evaluate, the method. For a real data set, we can first construct the tree from the real data and then generate more data sets under the null hypothesis by randomly permuting the affection status. Then, we can assess the significance of the markers in the tree of the real data on the basis of the distribution from the permutation data sets. We have used this technique effectively before [14].

## Competing interests

The authors declare that there are no competing interests.

## Authors’ contributions

YJ and HZ designed the study and carried out the data analysis. YJ, JSB, and HZ drafted the manuscript. JSB, RC, YH, and EN participated in data analysis. All authors read and approved the final manuscript.

## References

[B1] Ji W Foo JN O’Roak BJ Zhao H Larson MG Simon DB Newton-Cheh C State MW Levy D Lifton RP Rare independent mutations in renal salt handling genes contribute to blood pressure variation Nat Genet 2008 40 592 599 10.1038/ng.118 18391953 PMC3766631

[B2] Manolio T Collins F Cox N Goldstein D Hindorff L Hunter D McCarthy M Ramos E Cardon L Chakravarti A Finding the missing heritability of complex diseases Nature 2009 461 747 753 10.1038/nature08494 19812666 PMC2831613

[B3] Nejentsev S Walker N Riches D Egholm M Todd JA Rare variants of *IFIH1*, a gene implicated in antiviral responses, protect against type 1 diabetes Science 2009 324 387 389 10.1126/science.1167728 19264985 PMC2707798

[B4] Dering C Pugh E Ziegler A Statistical analysis of rare sequence variants: an overview of collapsing methods Genet Epidemiol 2011 X suppl X X X 10.1002/gepi.20643 PMC3277891 22128052

[B5] Li B Leal S Methods for detecting associations with rare variants for common diseases: application to analysis of sequence data Am J Hum Genet 2008 83 311 321 10.1016/j.ajhg.2008.06.024 18691683 PMC2842185

[B6] Madsen B Browning S A groupwise association test for rare mutations using a weighted sum statistic PLoS Genet 2009 5 e1000384 10.1371/journal.pgen.1000384 19214210 PMC2633048

[B7] Morris A Zeggini E An evaluation of statistical approaches to rare variant analysis in genetic association studies Genet Epidemiol 2010 34 188 193 10.1002/gepi.20450 19810025 PMC2962811

[B8] Wigginton JE Cutler DJ Abecasis GR A note on exact tests of Hardy-Weinberg equilibrium Am J Hum Genet 2005 76 887 893 10.1086/429864 15789306 PMC1199378

[B9] Purcell S Neale B Todd-Brown K Thomas L Ferreira MA Bender D Maller J Sklar P de Bakker PI Daly MJ PLINK: a tool set for whole-genome association and population-based linkage analysis Am J Hum Genet 2007 81 559 575 10.1086/519795 17701901 PMC1950838

[B10] Dasgupta A Sun YV Konig IR Bailey-Wilson JE Malley J A brief review of regression-based and machine learning methods in genetic epidemiology: the GAW17 experience Genet Epidemiol 2011 X suppl X X X 10.1002/gepi.20642 PMC3345521 22128059

[B11] Zhang H Singer B Recursive partitioning in the health sciences 1999 New York, Springer

[B12] Benjamini Y Hochberg Y Controlling the false discovery rate: a practical and powerful approach to multiple testing J R Stat Soc Ser B Methodol 1995 57 289 300

[B13] Almasy LA Dyer TD Peralta JM Kent JW Jr. Charlesworth JC Curran JE Blangero J Genetic Analysis Workshop 17 mini-exome simulation BMC Proc 2011 5 suppl 9 S2 22373155 10.1186/1753-6561-5-S9-S2 PMC3287854

[B14] Chen X Liu C-T Zhang M Zhang H A forest-based approach to identifying gene and gene-gene interactions Proc Natl Acad Sci USA 2007 104 19,199 19,203 10.1073/pnas.0709868104 PMC2148267 18048322

